# Case Report: Endoscopic resection of giant colonic pedunculated polyps in patients with AIDS using a novel nylon rope and titanium clip technique

**DOI:** 10.3389/fonc.2025.1566005

**Published:** 2025-05-14

**Authors:** YongXiang Xie, YongLi Zheng, Qian Tang, DongMei Wang

**Affiliations:** ^1^ Department of Endoscopy Center, Public Health Clinical Center of Chengdu, Chengdu, Sichuan, China; ^2^ Science and Education Division, Public Health Clinical Center of Chengdu, Chengdu, Sichuan, China

**Keywords:** novel nylon rope, titanium clip technique, endoscopic resection, giant colonic pedunculated polyps, AIDS

## Abstract

The three patients with AIDS were all male, aged between 56 and 67 years. One also had liver cirrhosis. They presented to the Chengdu Public Health Clinical Medical Center with a history of several months of blood in the stool. Colonoscopy revealed large pedunculated polyps in the sigmoid colon, with the largest measuring 5 cm and the smallest 3.5 cm. The polyps nearly completely obstructed the lumen. The long, thick stems were freely mobile within the narrow lumen, making it difficult to capture sufficient tissue with the inner ring of the forceps. The entire colon was examined with the aid of a transparent cap. Once the polyp was located, fecal water and residue surrounding it were removed. The polyp surface was then examined using white light and narrow-spectrum light to assess its structure. A biopsy was performed when cancer was suspected. During the procedure, we innovatively used a Harmony Clamp (ROCC-D-26-195; Micro Tech, Nanjing, China) to assist the nylon rope (HX-400U-30; Olympus, Tokyo, Japan) in pre-treating the pedicle root. First, the nylon rope was preloaded into the forceps channel of the enteroscope (EC-550L; SonoScape, Shenzhen, China), and the pedicle was ligated. When the polyp head turned deep purple and the stalk became pale, it confirmed that the blood supply to the mass had been fully blocked. A Harmony Clamp was then inserted into the intestinal cavity through the forceps channel, and the nylon rope was secured around the base of the polyp. The electric snare (VDK-SD-23-230-25-A1; Vedkang, Jiangsu, China) was placed between the clamp and the polyp, at least 0.5 cm from the mass, followed by high-frequency electroresection. Postoperatively, the wound appeared white with no bleeding or perforation. The operation time for all three patients was between 5 and 7 minutes, and there were no complications such as bleeding, perforation, or abdominal pain during or after the procedure. Follow-up colonoscopy 1 to 3 months later showed scar formation in the surgical area and no recurrence. Pathological analysis revealed that two cases were tubular-villous adenomas, and one case was a tubular-villous adenoma with focal high-grade intraepithelial neoplasia and mucosal carcinoma, with negative horizontal and vertical margins.

## 1 Introduction

**Figure 1:**
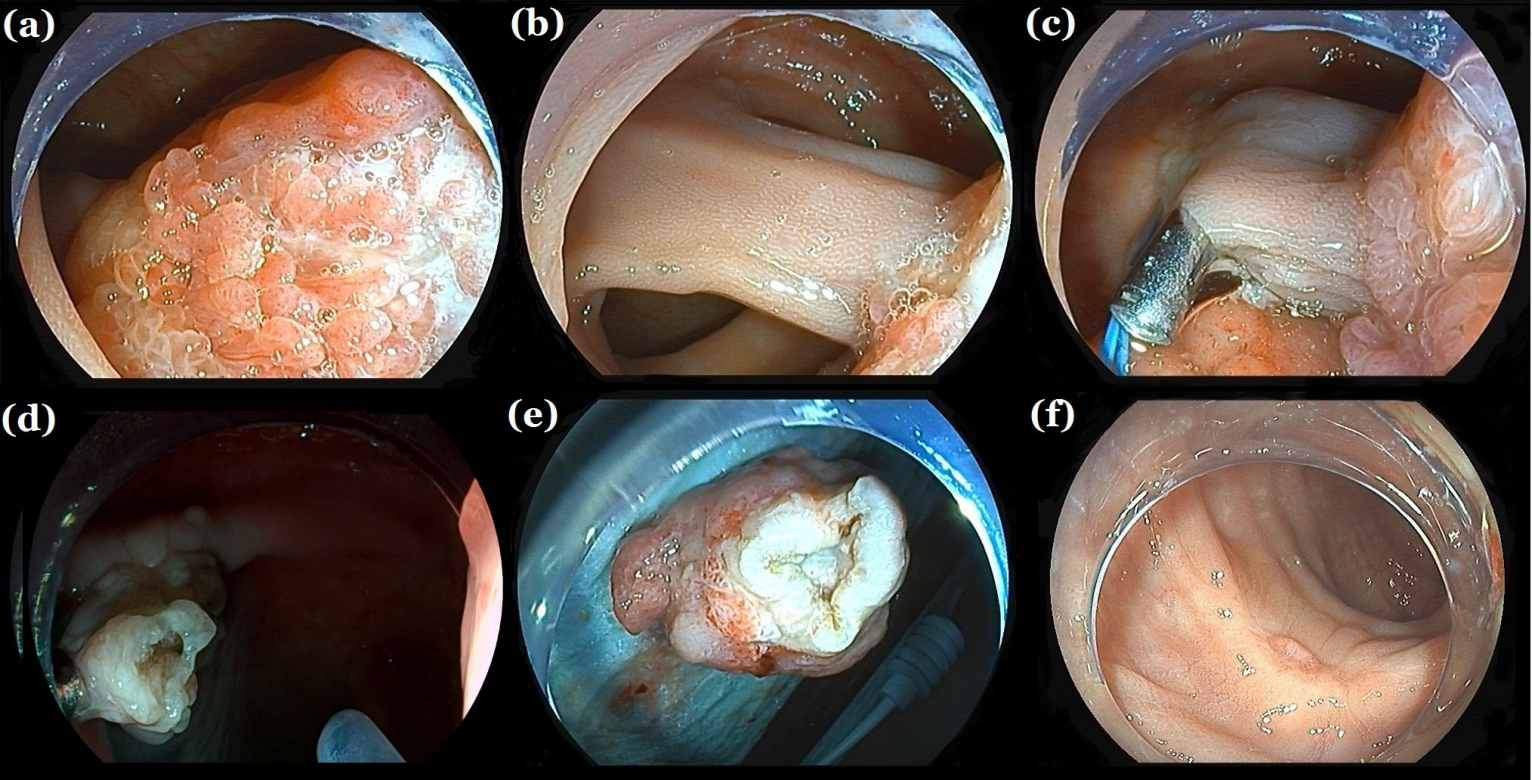
Endoscopic resection. **(a, b)** A large colon pedunculated polyp nearly completely obstructing the lumen. **(c)** Titanium clips assisting nylon cord ligation at the thick peduncle root. **(d)** No bleeding or perforation at the wound site after polypectomy. **(d)** Follow-up colonoscopy 1 month later showing scar formation. **(e)** giant colorectal polyps. **(f)** A re-examination of the colonoscopy 1 month later showed scar formation.

**Figure 2:**
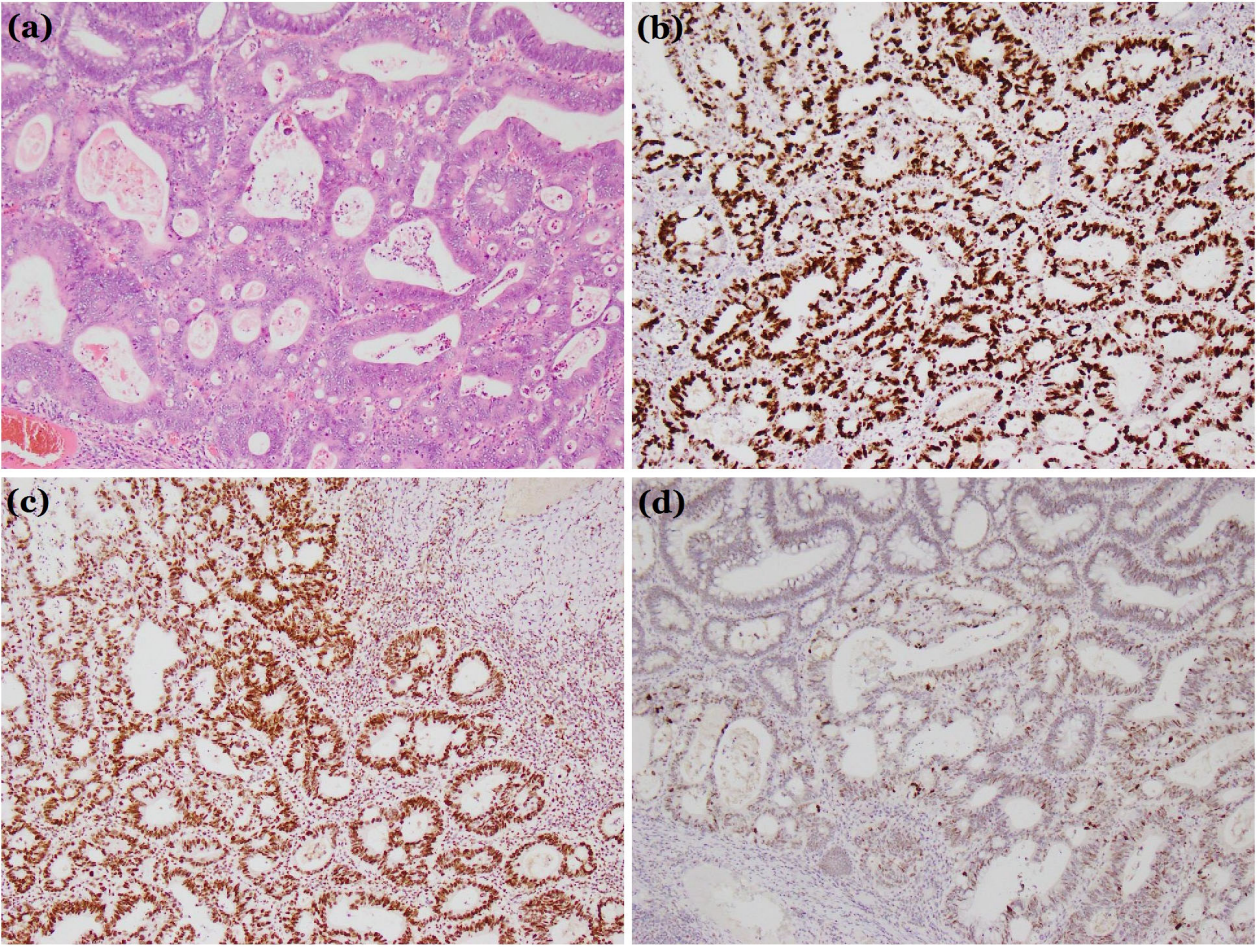
Histological diagnosis. **(a)** Hematoxylin and eosin stain (×100). **(b-d)** Immunohistochemical examination: **(b)** Ki67 (+). **(c)** MSH2 (+). **(d)** P53 (+). These findings confirmed the tumor was a villous-tubular adenoma with high-grade dysplasia and focal intramucosal carcinoma.

In contemporary clinical practice, a variety of endoscopic resection techniques for thick pedunculated polyps have been developed. These methods include traditional endoscopic mucosal resection (EMR), titanium clip occlusion of the root, and nylon rope ligation of the root. Thick pedunculated polyps often have relatively large blood vessels (1). Colon polyps can be classified into adenomatous, inflammatory, hyperplastic, and hamartomatous types based on their pathological features (2). Among these, adenomatous polyps carry the highest risk of malignant transformation (3, 4). Colorectal cancer is among the most prevalent cancers in both women and men (5, 6). In terms of morphology, polyps can be categorized as broad-based, sub-pedunculated, or long-pedunculated. For broad-based polyps smaller than 1 cm, cold resection with a snare is the primary method. Titanium clips are only suitable for endoscopic resection of long-pedunculated polyps with a diameter around 5 mm. Polyps with thick peduncles greater than 1 cm pose a risk of massive bleeding due to incomplete blood supply blockage. The incidence of complications in traditional polyp resection Endoscopic Mucosal Resection (EMR) technology is relatively high, with a bleeding rate of up to 7.1% (7–9), While nylon rope ligation can effectively block the blood supply, it may slip off prematurely after surgery, leading to delayed bleeding. This report presents an innovative approach: first, the root is ligated with a nylon rope, then the nylon rope is fixed with a titanium clip, followed by high-frequency electroresection (**Figures 1**, **2**). This combined method leverages the strengths of both techniques, compensating for their individual limitations. The nylon rope effectively blocks the blood supply, while the titanium clip secures the nylon rope at the root, preventing premature slippage (**Figure 1c**). It is worth noting that the roots of large and long pedunculated polyps often have huge nourishing blood vessels to supply the growth of the polyps, so the surface of the polyps will turn red (**Figure 1a, b**). After the roots are ligated with nylon ropes, the blood vessels are blocked, and the surface of the polyps will immediately appear bruised. During the surgery, it can be judged that the blood supply has been blocked, and then surgery can be performed, and reducing the risk of delayed bleeding.

This study has several limitations. First of all, although all three cases were successfully treated with this method to remove giant colonic pedunculated polyps, the operation time was brief, the surgical field remained clear, and no bleeding or perforation occurred during the procedure. There were no complications, such as delayed bleeding or perforation, postoperatively. The resected specimens showed negative vertical and horizontal margins (**Figures 1d–f**), eliminating the need for additional surgical intervention and reducing the mental and economic burden on the patients. However, the number of cases and scope of application of this method are limited, and further expansion of its scope is needed, such as further validation in populations with underlying diseases, different immune function states, etc., to provide data support for the implementation and further promotion of this method. In addition, As of the submission time of this manuscript, all cases have been followed up for at least 1 year, and no tumor recurrence has occurred in any of the 3 cases. However, a longer follow-up time may be needed clinically to evaluate the efficacy of surgery and the recurrence of tumors.

In conclusion, titanium-clip-assisted nylon rope pre-treatment of the polyp root in the endoscopic resection of large colonic thick pedunculated polyps is both safe and effective. The procedure offers a short operation time, a clear surgical field, and no bleeding or perforation during or after the operation. It is a technique worthy of clinical adoption, particularly in patients with infectious diseases.

## Data Availability

The raw data supporting the conclusions of this article will be made available by the authors, without undue reservation.
